# Reduced immune responses to hepatitis B primary vaccination in obese individuals with nonalcoholic fatty liver disease (NAFLD)

**DOI:** 10.1038/s41541-020-00266-4

**Published:** 2021-01-11

**Authors:** Shivali S. Joshi, Rachelle P. Davis, Mang M. Ma, Edward Tam, Curtis L. Cooper, Alnoor Ramji, Erin M. Kelly, Saumya Jayakumar, Mark G. Swain, Craig N. Jenne, Carla S. Coffin

**Affiliations:** 1 Department of Medicine, Cumming School of Medicine, University of Calgary, Calgary, AB Canada; 2 Department of Microbiology, Immunology & Infectious Diseases, Cumming School of Medicine, University of Calgary, Calgary, AB Canada; 3 University of Alberta, Edmonton, AB Canada; 4 Pacific Gastroenterology Associates, Vancouver, BC Canada; 5 Ottawa Hospital Research Institute, Ottawa, ON Canada; 6 Division of Gastroenterology, University of British Columbia, Vancouver, BC Canada; 7 Present Address: University of British Columbia, Vancouver, BC Canada

**Keywords:** Biomarkers, Diseases, Immunology, Microbiology

## Abstract

Obesity and cirrhosis are associated with poor hepatitis B virus (HBV) vaccine responses, but vaccine efficacy has not been assessed in nonalcoholic fatty liver disease (NAFLD). Sixty-eight HBV-naïve adults with NAFLD were enrolled through the Canadian HBV network and completed three-dose HBV or HBV/HAV vaccine (Engerix-B^®^, or Twinrix^®^, GlaxoSmithKline). Anti-HBs titers were measured at 1–3 months post third dose. In 31/68 subjects enrolled at the coordinating-site, T-cell proliferation and follicular T-helper cells (pTFH) were assessed using PBMC. Immune response was also studied in NAFLD mice. NAFLD patients were stratified as low-risk-obesity, BMI < 35 (*N* = 40) vs. medium-high-risk obesity, BMI > 35 (*N* = 28). Anti-HBs titers were lower in medium/high-risk obesity, 385 IU/L ± 79 vs. low-risk obesity class, 642 IU/L ± 68.2, *p* = 0.02. High-risk obesity cases, *N* = 14 showed lower vaccine-specific-CD3+ CD4+ T-cell response compared to low-risk obesity patients, *N* = 17, *p* = 0.02. Low vaccine responders showed dysfunctional pTFH. NAFLD mice showed lower anti-HBs levels and T-cell response vs. controls. In conclusion, we report here that obese individuals with NAFLD exhibit decreased HBV vaccine-specific immune responses.

## Introduction

The implementation of universal childhood hepatitis B virus (HBV) vaccination in >200 countries has led to a significant decline in the global incidence of chronic hepatitis B and hepatocellular carcinoma (HCC), and has been hailed as the first vaccine known to prevent cancer^1^. All available HBV vaccines contain the major HBV surface antigen (HBsAg) and induce antibody and T cell responses in >85% of immunocompetent adults^2–4^. The Centres for Disease Control and Prevention recommends the HBV vaccine to all individuals with chronic liver disease (i.e., persons with hepatitis C virus (HCV) infection, diabetes, autoimmune hepatitis, alcohol, and nonalcoholic fatty liver disease (NAFLD))^5^.

Over the last 2 decades, the obesity epidemic has contributed to the development of the metabolic syndrome and NAFLD^6–8^. Obesity leads to a chronic inflammatory state and negatively affects immunity as evidenced by higher rates of vaccine failure, immunity against foreign pathogens, and infection complications^9^. In general, there is limited knowledge regarding the impact of obesity and NAFLD on immune functions per se. Studies in animal models suggest altered lymphocyte responsiveness to mitogens, dysregulated cytokine expression, decreased macrophage, natural killer cell, and dendritic cell function to pathogens^10,11^. In general, obesity can disrupt overall immune system integrity and lead to alterations in leukocyte development, migration, and diversity. Although HBV vaccination is strongly recommended in all individuals with chronic liver disease, there is limited data on HBV vaccine responses in adults with obesity and NAFLD. Moreover, most studies only focussed on B-cell immunity (i.e., antibody to HBV surface (anti-HBs) responses) to the vaccine. Some studies have found that obesity was associated with poor response to HBV vaccine in preadolescents and in adults with body mass index (BMI) >30 kg/m^2^ compared to non-obese individuals^12,13^. Reduced anti-HBs seroconversion rates were also found in adults with diabetes and renal disease compared to healthy adults^14,15^. Although one study found a high prevalence of HBV vaccine unresponsiveness in ~70% of pediatric patients with NAFLD^16^, another reported no difference in vaccine response rates in children with NAFLD compared to healthy controls^17^.

We conducted a prospective interventional study to assess the efficacy of HBV vaccination in HBV-naïve adults with NAFLD in Canada, a low HBV endemic country that did not implement universal childhood immunization until the mid 1990s^18^. In this study, we assessed anti-HBs titers, ex vivo B- and T-cell responses, and the role of a special subset of CD4+ T cells (i.e, peripheral T follicular helper cells, pTFHs) involved in generation and maintenance of memory B and plasma cells following completion of three-dose HBV vaccine series (i.e., EngerixB^®^ or Twinrix^®^). Moreover, we provide supportive parallel HBV immunization data from a unique high-fat diet (HFD)-induced mouse model of NAFLD.

## Results

### Summary of baseline demographic and clinical data

**Fig. 1:**
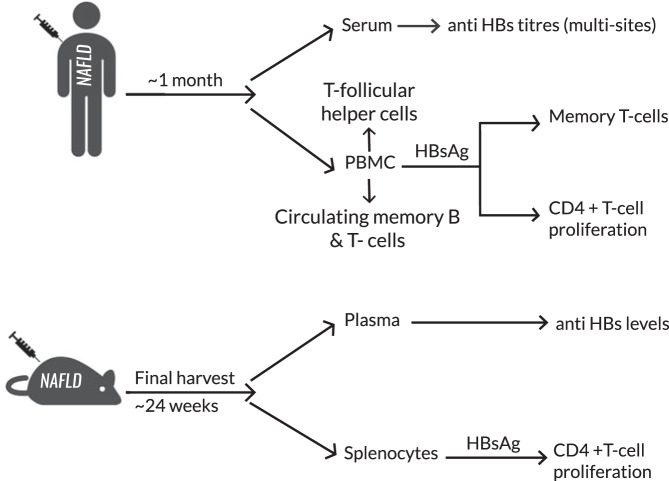
Schematic depicting study design and experiments. NAFLD nonalcoholic fatty liver disease, HBsAg hepatitis B surface antigen, Anti-HBs antibody to hepatitis B surface antigen, PBMC peripheral blood mononuclear cells.

In total, 386 patients were screened of which 356 were excluded, usually due to prior HBV exposure or vaccination (*N* = 193) or age (*N* = 127). Three withdrew from the study or were lost to follow-up. Overall, 68 HBV naive NAFLD adults completed the standard HBV primary vaccination and stratified as BMI < 35 (low-risk obesity) or BMI > 35 (medium high-risk obesity) based on Health Canada nomogram^19^. Figure 1 describes the experimental design of this multisite, phase IV, open-label interventional study. The clinical characteristics of enrolled patients are included in Table 1. The overall median age was 50 years, 35 females, 78% (53/68) Caucasian,16% (11/68) diabetic, 40 had BMI < 35 vs. 28 with BMI > 35. In 31 enrolled at the coordinating site, ex vivo B- and T-cell responses (17 with BMI < 35 and 14 with BMI > 35) (Table 2) were also assessed.

**Table 1:** Baseline characteristics of vaccinated study participants.

Obesity class	Number (*N* = 68) [M/F]	Ethnicity	Median age (years)	Median BMI* in kg/m^2^ (range)	Median TE scores^#^ in kPa (range)	Median ALT in U/L (range)	Diabetes/dyslipidemia/hypertension
BMI < 35	40 [22M/18F]	28 Caucasian 7 Asian 1 Hispanic 1 Middle Eastern 3 mixed ethnicity	50 (24–64)	29.6 (21.8–34.7)	5.3 (2.5–27.4)	47 (9–120)	7/10/12
BMI > 35	28 [11M/17F]	25 Caucasian 3 Asian	50 (24–62)	37.9 (35–52.3	6.4 (3.8–50.6)	38 (14–205)	4/9/16

BMI body mass index, TE transient elastography, ALT alanine transaminase.

* BMI range for obesity by Health Canada and WHO:

Obesity Class-I (low-risk obesity): 30–34.9.

Obesity Class-II and III (high-risk obesity): 35–39.9 and ≥40, respectively.

BMI range for Asians^48^:

Obesity Class-I (low-risk obesity): 25–29.9.

Obesity Class II and III (high risk obesity): 30–34.9 and ≥35, respectively.

**Table 2:** Clinical characteristics of patients with hepatitis B vaccine specific B- and T-cell responses analyzed.

Obesity class	Number (*N* = 31) [M/F]	Ethnicity	Median age (years)	Median BMI in kg/m^2^ (range)	Median TE scores, kPa (range)	Median ALT, U/L (range)	Diabetes/dyslipidemia/hypertension
BMI < 35	17 [7M/ 10F]	14 Caucasian 1 Asian 2 mixed ethnicity	52 (37–61)	30.8 (21.8–34.7)	4.6 (2.5–8.6)	40 (15–77)	3/6/4
BMI > 35	14 [6M/8F]	13 Caucasian 1 Asian	56 (24–62)	37.9 (35–52.3)	7.7 (3.8–50.5)	27 (14–124)	1/3/8

BMI body mass index, TE transient elastography.

### Anti-HBs titers in adults with NAFLD after completion of three-dose HBV vaccine series negatively correlated with BMI

**Fig. 2:**
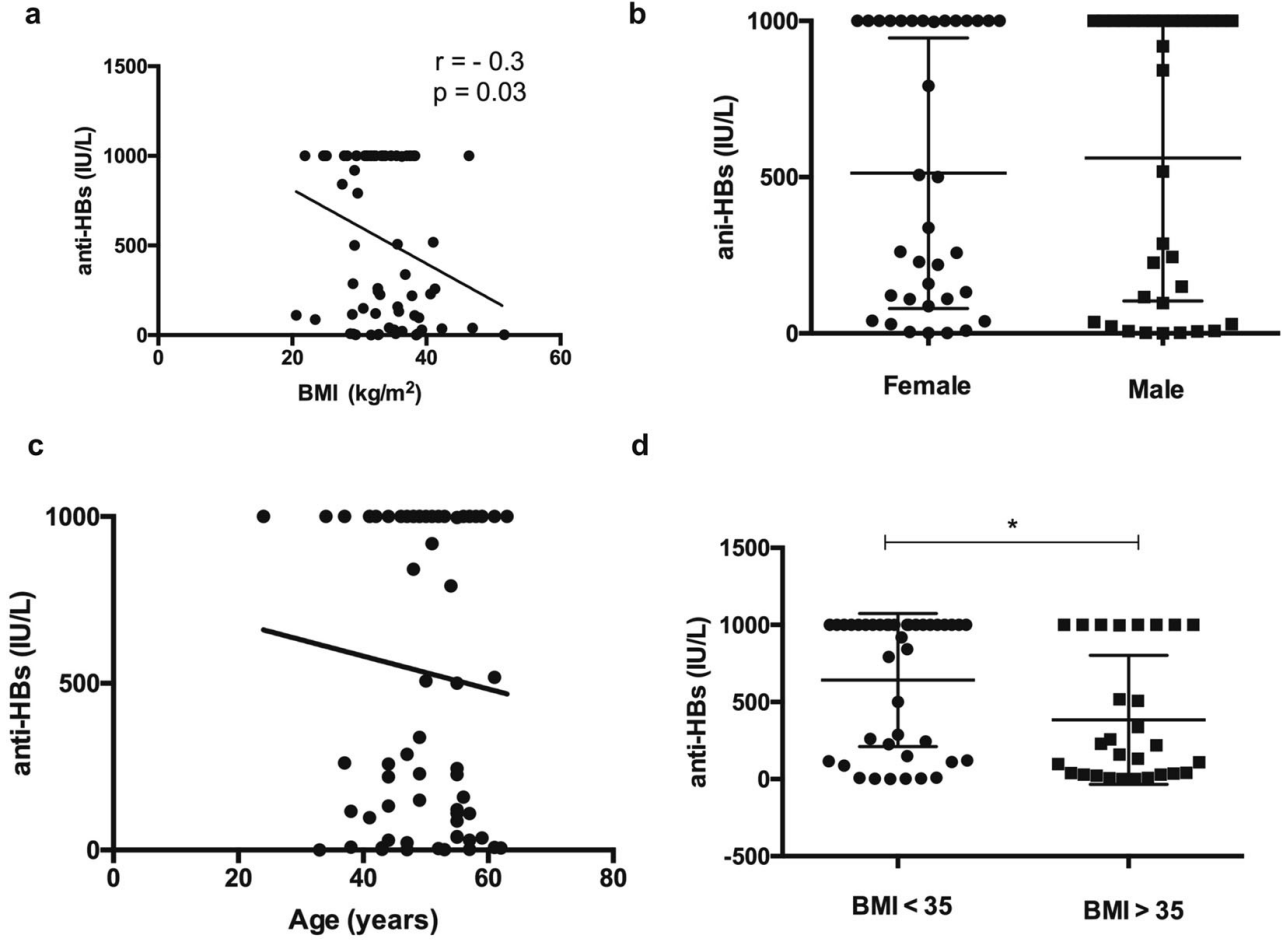
Anti-HBs levels in NAFLD patients. **A** Spearman’s rank correlation between anti-HBs levels with BMI, in *N* = 68 vaccinated NAFLD patients. The data show a moderate negative correlation between BMI and anti-HBs titers (Spearman’s Rank correlation co-efficient *r* = −0.3, *p* = 0.03). **B** Anti-HBs titers were comparable between males and females (*p* = 0.7, Mann–Whitney *U* test). **C** No association was noted between age and anti-HBs levels. **D** Anti-HBs levels in low and high-risk obesity patients with NAFLD. Anti-HBs levels were significantly lower (*p* = 0.02) in the high risk obesity group vs. low-risk obesity group NAFLD patients (Mann–Whitney *U* test). Data is presented as mean ± standard deviation.

In total, 85% (58/68) of study participants developed protective anti-HBV surface levels (anti-HBs > 10 IU/L) (Table 3). HBV vaccine nonresponse (anti-HBs < 10 IU/L) was found in 10/68 NAFLD patients. The rates of vaccine non-response were similar in low- vs. high-risk obesity class patients with NAFLD (i.e., 15% (6/40) of patients with BMI < 35 and 14.2% (4/28) of patients with BMI > 35 were nonresponders) (Table 4). Poor response to the vaccine was not related to age, gender, smoking status, metabolic syndrome (diabetes, dyslipidemia, and hypertension), and fibrosis stage (including patients who underwent liver biopsy, Supplementary Table 1). All the vaccine nonresponders had increased waist circumference indicating abdominal obesity and risk of obesity related health problems based on Health Canada recommendations (i.e., ≥102 cm for males and ≥82 cm for females)^20^ (Table 4). Interestingly, a negative correlation (*r* = −0.3, *p* = 0.03) was observed between anti-HBs and BMI (Fig. 2A). In addition, humoral vaccine response was not associated with age and sex (Fig. 2B, C).

**Table 3:** Anti-HBs titers in low- and high-risk obesity class patients.

Anti-HBs levels (IU/L)	Low-risk obesity, BMI < 35, *N* = 40	High-risk obesity, BMI > 35, *N* = 28
1000 (high responders)	21 (52.5%)	7 (25%)
100–999 (normal responders)	12 (30%)	10 (35.7%)
10–99 (low responders)	1 (2.5%)	7 (25%)
<10 (nonresponders)	6 (15%)	4 (14.2%)

*classification of vaccine responders and non-responders as per Liu et al., 2017^22^.

**Table 4:** Clinical characteristics of HBV vaccine nonresponders in the study (i.e., anti-HBs titers < 10 IU/L).

Patient ID	Age (years)	Ethnicity	Gender	BMI	Anti-HBs (IU/L)	WC	TE (kPa)
0005-1086	58	Asian	F	29.4	2	102	8.5
0001-8655	43	Hispanic	M	32.8	2	96	7.6
0006-7710	34	Caucasian	M	38.4	0	140	7.9
0011-9100^$^	44	Caucasian	M	29	7	95	16.9
0002-2081^$^	53	Caucasian	F	52.3	1.6	142	17.5
0015-4123	50	Caucasian	M	38.7	6.4	128	8.1
0016-5139**	38	Caucasian	F	28.6	8.4	94	4.3
0019-9044	61	Caucasian	M	35.4	8	113.5	12.1
0021-2898^#^	52	Caucasian	F	32.8	4.7	104	8.6
4139-0001^$^	47	Caucasian	F	31.7	1	40	27.9

BMI body mass index, TE transient elastography, WC waist circumference, WC cut offs: ≥82 cm for women and ≥102 cm for men.

None of the patients had a history of smoking

^#^Diabetic.

**Hypertension.

^$^Compensated cirrhosis.

### NAFLD patients with BMI > 35 show weaker humoral immune responses to hepatitis B vaccine compared to those with BMI < 35

Overall, NAFLD patients with BMI > 35 showed significantly lower mean anti-HBs levels compared to low-risk obesity class patients with BMI < 35. The mean anti-HBs levels in patients with BMI > 35 were 385 IU/L ± 79 compared to 642 IU/L ± 68.2 in patients with BMI < 35 (*p* = 0.02; Fig. 2D).

### Assessment of total memory B cells

Previous studies have shown that individuals who are nonresponders to the HBV vaccine (anti-HBs < 10 IU/L) show a strong anamnestic response to a booster dose suggesting an effect on memory B cells. Analyses of PBMC were performed in subjects (*N* = 31/68) enrolled at the coordinating site. The proportion of total memory B cells CD45+ CD19+ CD27+ in PBMC from NAFLD patients was comparable between patients with BMI > 35 vs. BMI < 35, *p* > 0.05 (Supplementary Fig. 1).

### Assessment of memory T cells and HBsAg-specific-proliferation of CD3 + CD4+ T_H_ cells

**Fig. 3:**
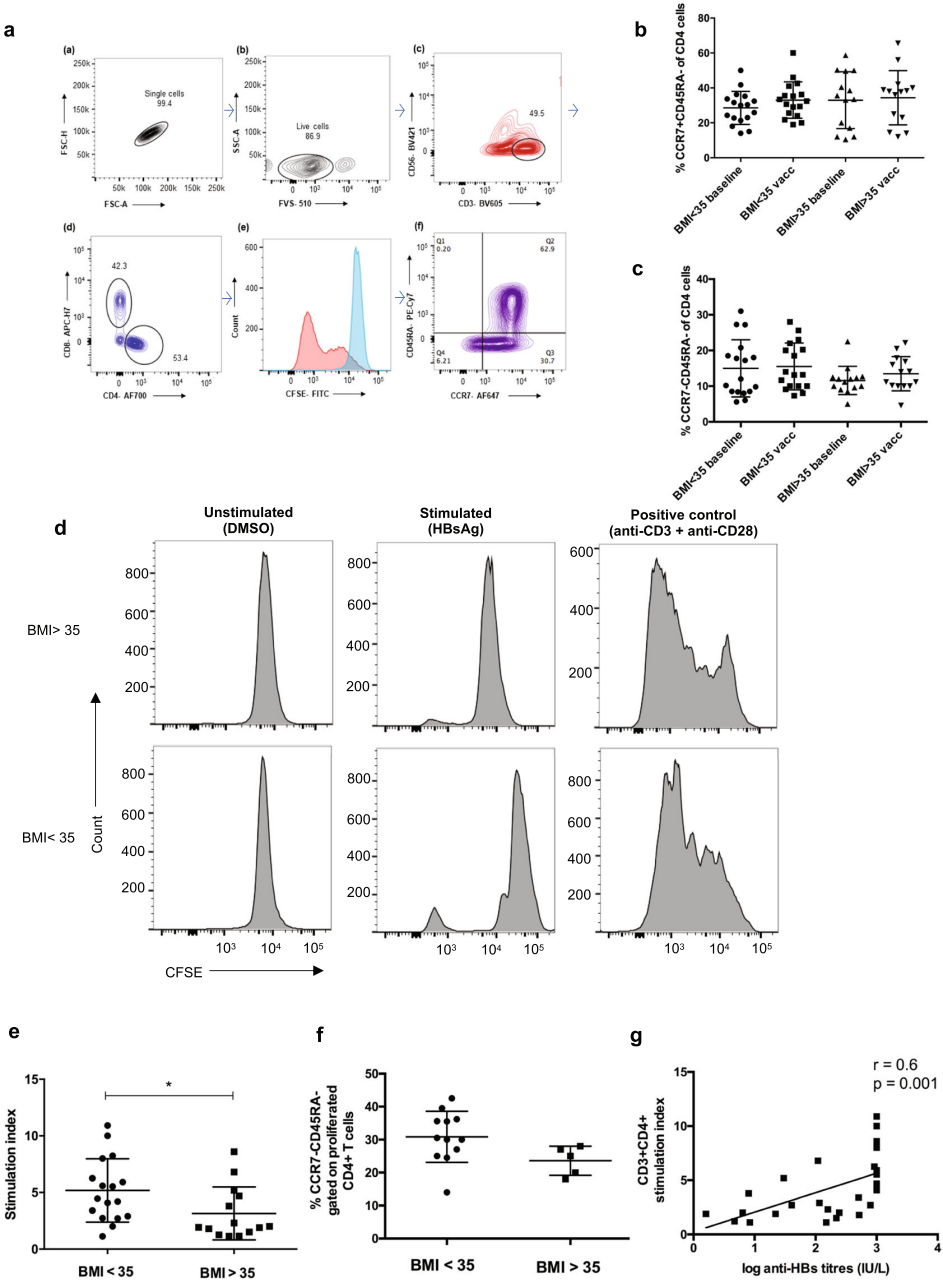
Immunophenotyping of CD4+ memory T cells and proliferation. **A** Gating strategy for T cells. **B** Proportion of circulating central memory cells (T_CM_, CCR7+ CD45RA−). **C** Proportion of circulating effector memory cells (T_EM_, CCR7− CD45RA−) gated on live CD3+ CD56− CD4+ cells at baseline and after vaccination in NAFLD patients. The data show no differences in the percentage of the cells at baseline and post vaccination between low and high-risk obesity NAFLD cases (Krushkal–Wallis test). **D** Representative CFSE proliferation assays from a low (showed proliferation) and high risk patient (who did not show proliferation) using PBMC. Every sample was cultured in triplicates with DMSO controls, HBsAg stimulated cells (5 µg) for ~8 days and positive control anti-CD3 (1 µg/mL) + anti-CD28 (5 µg/mL) beads for 4 days. **E** Stimulation index (SI) in low risk vs. high-risk cases: high risk obesity class NAFLD patients showed weaker T cell proliferation compared to low-risk patients, *p* = 0.02 (Mann–Whitney *U* test). **F** Proportion of effector memory cells among the proliferated CD4+ T cells: T_EM_ frequencies between 12 low-risk and 5 high-risk obesity patients that showed HBsAg specific T-cell proliferation were comparable (Mann–Whitney *U* test). Data are presented as mean ± standard deviation. **G** Correlation between anti-HBs levels, IU/L and CD4+ T cell stimulation index: Spearman’s Rank correlation co-efficient = 0.6, *p* = 0.001. CFSE carboxyfluorescein diacetate succinimidyl ester, PBMC peripheral blood mononuclear cells.

Long term T cell-mediated protection depends on the generation of a pool of memory cells to protect against future pathogen challenge. The two types of memory T cells central to a vaccine response are central memory T cells (T_CM_), having a reactive function (similar to memory B cells), and effector memory cells (T_EM_) that are protective (similar to antibodies)^21^. The gating strategy for memory T cells is shown in Fig. 3A. The proportion of circulating T_EM_ and T_CM_ cells between NAFLD adults with BMI < 35 and BMI > 35 at baseline and after vaccination showed no significant difference (Fig. 3B, C). CD4+ T cells showed HBsAg-specific-proliferation in 12/17 (70.5%) cases in patients with BMI < 35 group compared to 5/14 (35.7%) with BMI > 35 (Fig. 3D, E). In addition, high-risk obesity NAFLD patients (mean SI 3.1 ± 0.62) showed a reduced proliferation compared to low-risk cases (mean stimulation index (SI) 5.1 ± 0.68), *p* = 0.02. Further, gating of CD3+ CD4+ CFSE low cells revealed comparable frequencies of T_EM_ cells between both groups (Fig. 3F). In 2/17 cases with BMI < 35, and 1/13 BMI > 35 we found antigen specific proliferation of CD8+ cells (Supplementary Fig. 2). This was unexpected given that CD8+ T cell responses are difficult to induce by proteins and these responses are likely due to cross presentation of antigen to CD8+ T cells.

### Correlation between HBsAg-specific-T-cell response and humoral response

A direct correlation has been reported previously between serological and T-cell responses^22^. We found a positive correlation between serum anti-HBs levels and CD4+ T cell SI in 31 patients (Fig. 3G).

### Serum CXCL13 and IL-21 and peripheral T-follicular helper cells (pTFHs) in NAFLD patients at baseline and post vaccination

**Fig. 4:**
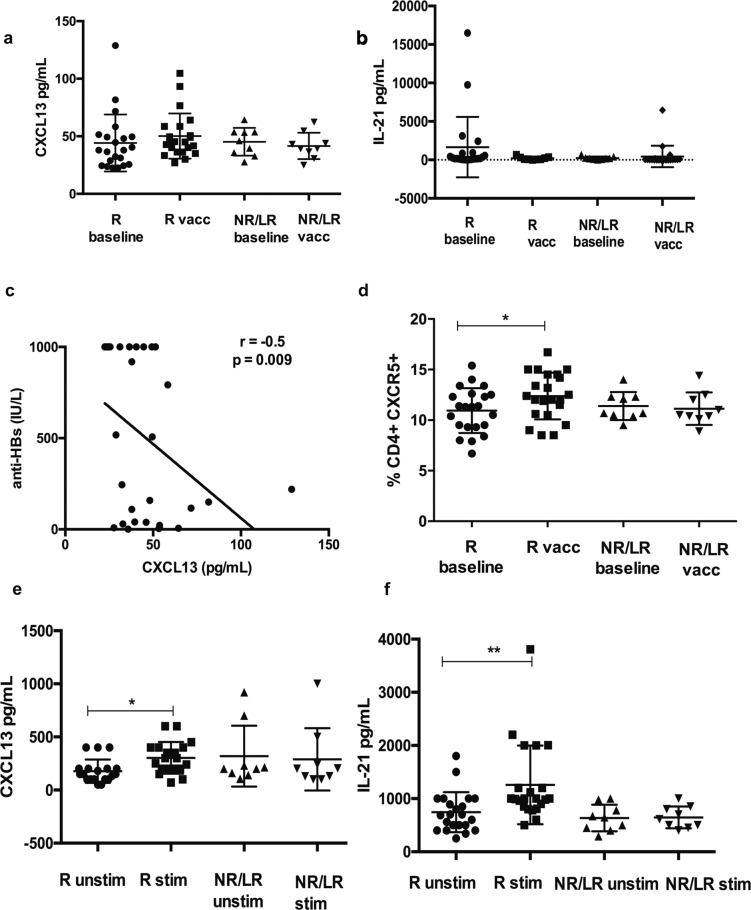
Peripheral follicular helper T (pTFH) cell analysis. **A** Serum CXCL13 **B** serum IL-21 levels in pg/mL at baseline and post vaccination in 31 NAFLD patients. **C** Spearman’s rank correlation between baseline CXCL13 and anti-HBs levels in NAFLD, *r* = −0.5, *p* = 0.009. **D** Frequency of pTFH in PBMC among NAFLD patients. **E** HBsAg specific CXCL13 production by pTFH cocultured with B cells. Sorting experiments were carried out in *N* = 19/21 responders and *N* = 9 non/low responders. Data are presented as mean ± standard deviation. R normal and high responders, anti-HBs > 100 IU/L, NR/LR nonresponders and low responders, anti-HBs < 100 IU/L, unstim unstimulated, stim stimulated.

Initially, we compared the pTFH cell response in NAFLD patients with BMI > 35 and BMI <35 at baseline and post vaccination and found no difference among the groups (Supplementary Fig. 3). We then grouped patients on the basis of antibody levels given that pTFH cells, a subset of CD4+ T cells are involved in generation and maintenance of memory B and plasma cells^23^. We evaluated two important effectors, serum CXCL13 and IL-21 levels and proportion of pTFH between non/low responders, anti-HBs < 100 IU/L and normal-high responders (anti-HBs > 100 IU/L). No differences were observed in the baseline and post vaccination cytokine levels in both the groups (Fig. 4A, B). However, we found a negative correlation between baseline serum CXCL13 levels and anti-HBs levels (Fig. 4C). Interestingly, post vaccination increase in pTFH was noted in only in the normal-high responders but not in the non/low responders (Fig. 4D).

### Antigen specific release of CXCL13 and IL-21 by sorted pTFHs

Coculture of pTFHs with B cells in the presence of HBsAg showed a significant antigen specific increase in both CXCL13 and IL-21 in the normal-high responders but not in the non-low responders suggesting dysfunctional pTFHs in this group (Fig. 4E, F).

### Anti-HBs antibody levels and T-cell responses in a mice model of NAFLD

**Fig. 5:**
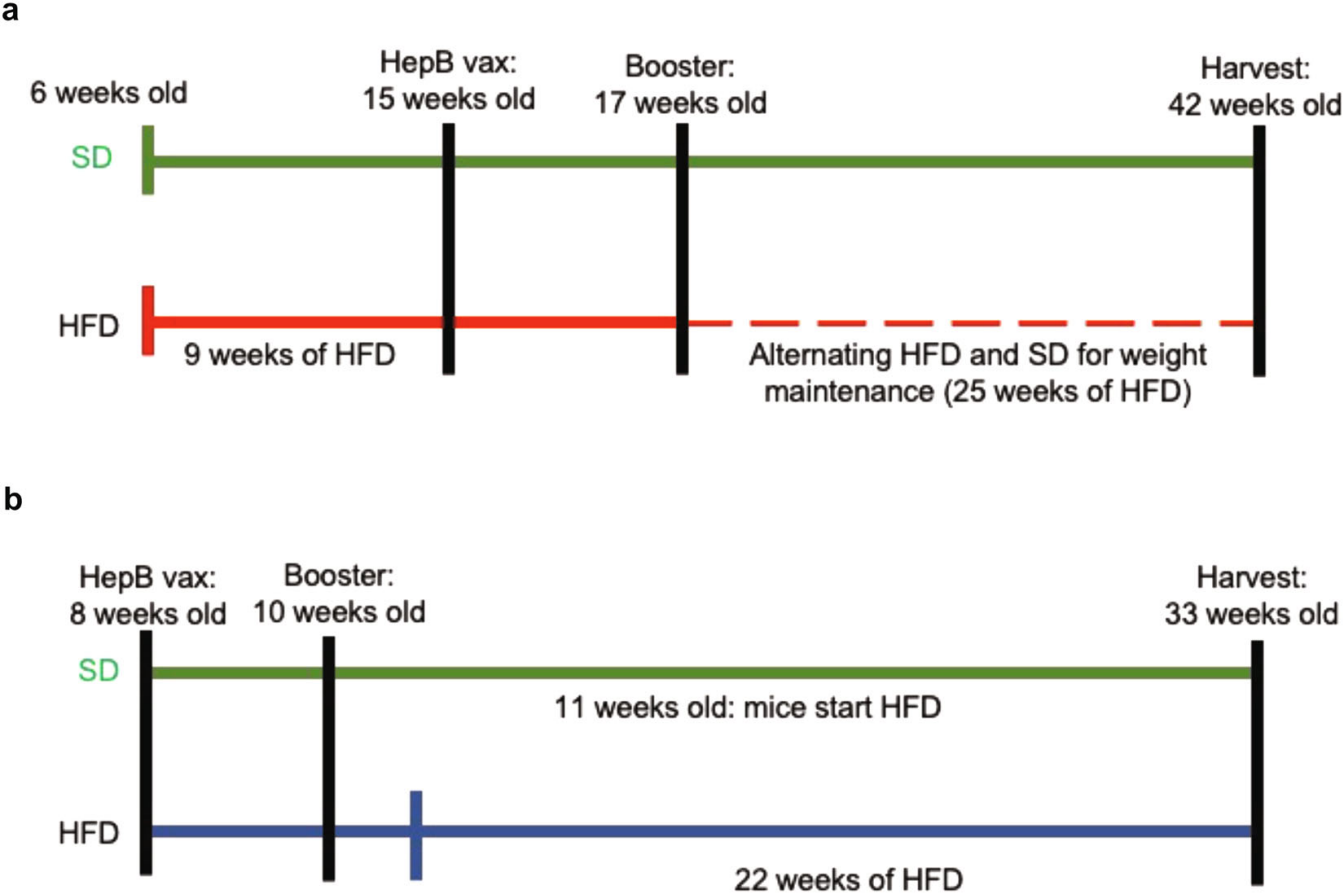
Mice model of NAFLD and hepatitis B vaccination. **A** Represents NAFLD→vaccination group (N→V), mice-fed high-fat diet (HFD) for 9 weeks and subsequently vaccinated. **B** Vaccination→NAFLD (V→N) mice receiving the vaccine at baseline and subsequently fed high-fat diet. HepB vax Engerix B vaccine, SD standard diet.

**Fig. 6:**
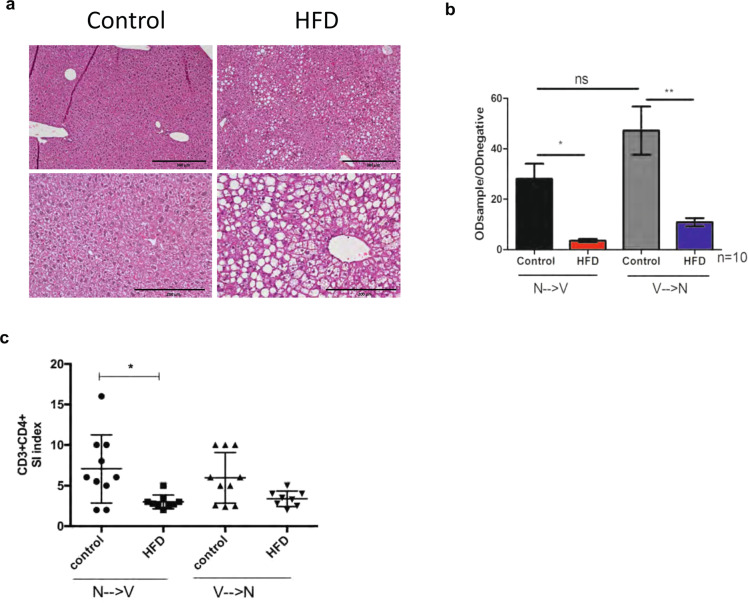
Antibody and T cell response in high-fat-diet (HFD)-induced mouse model. **A** Liver histology of control and HFD mice showing hallmarks of fatty liver disease. Greater than 66% steatosis was noted in zone 2 and 3. **B** Qualitative ELISA showing anti-HBs in NAFLD mice vs. controls. NAFLD mice showed significantly lower levels of anti-HBs than controls (*p* < 0.05, ANOVA test). **C** In vitro proliferation of CD3+ CD4+ splenocytes at ~day 5 in response to 5 µg HBsAg. Mice in the NAFLD→vaccination (N→V) group showed significantly lower proliferation than controls (*p* < 0.05, ANOVA). The same trend was observed in the Vaccination→NAFLD (V→N) group. Data are represented as mean ± standard deviation.

Mice models who were fed a HFD to induce NAFLD and given the HBV vaccine are depicted in Fig. 5. The liver histology of mice on HFD showed hallmarks of fatty liver disease (<66% steatosis in zone 2 and 3) confirming HFD-induced NAFLD (Fig. 6A). HFD mice both in group V→N (vaccinated before NAFLD was induced) and in N→V group (vaccine administered after NAFLD was induced by HFD) exhibited lower anti-HBs antibody levels than controls (*p* < 0.05) (Fig. 6B). The N→V group mice also showed a lower CD4+ T-cell response than normal mice and a similar trend was observed in V→N study mice compared to controls (Fig. 6C).

## Discussion

This novel interventional study was designed to evaluate the efficacy of hepatitis B vaccine using a standard dosing schedule in HBV naïve/ previously unvaccinated adults with a clinical diagnosis of NAFLD. Overall, this study determined that upon completion of the full vaccination regimen, using the approved recombinant hepatitis B vaccine, seroprotection was induced in ~85% of subjects. A similar rate of nonresponse (15–20% of patients with anti-HBs < 10 IU/L) to the HBV vaccine have been shown in persons with diabetes and inflammatory bowel disease^24,25^. Importantly, this reduced antibody response was more pronounced in high-risk obesity class NAFLD patients with BMI > 35 compared to individuals with a lower BMI < 35 at only ~1 month post primary vaccination. Similar to others, we noted a negative correlation between response to vaccination and BMI^26^. However, it is also noteworthy that 3 of 10 nonresponders in the study were cases with a BMI 25–30 (i.e., overweight but nonobese individuals). We found comparable rates of non-immunity (~15%) in both low- vs. high-risk obesity groups, and hence addresses the potential issue of sufficient needle length for immunization of obese individuals^27^. Older age at the time of vaccination (>60 years) has been associated with inadequate antibody response^28^. The median age in the group in our study was ~50 years and no correlation between age and humoral response was observed. Further, known factors for vaccine non-response such as liver fibrosis stage (i.e., cirrhosis), metabolic syndrome (i.e., diabetes, hypertension, and dyslipidemia), gender or smoking status were not associated with antibody response in the current study^29^.

Antibody response to hepatitis B vaccine involves both B and T cells. We analyzed T cell responses in 31 NAFLD patients and found reduced HBsAg-specific-CD4+ T cell response in patients with BMI > 35 compared to patients with BMI < 35. We noted a comparable frequency of T_EM_ among the proliferated cells in low- and high-risk obesity groups, however, we did not investigate the cytokine production by these cells. Prior studies found intact T cell responses in healthy individuals immunized with the recombinant HBV vaccine and the traditional plasma derived vaccine in the absence of antibodies^30^. In obesity, cell death and enlarged adipocytes occurs due to a constant pro-inflammatory state. Additionally, there is a reduced proportion of CD4+ T cells, which are activated by nonconventional means involving leptin and result in sub-optimal CD4+ T cell differentiation and proliferation in response to antigens^31,32^. Moreover, studies in NAFLD mice models suggest decreased antigen processing and presentation in splenic dendritic cells compared to controls in association with an impaired T cell response^33^.

Dysfunctional pTFH cells have been reported in nonresponders with anti-HBs < 10 IU/L to the HBV vaccine in healthy subjects and also in influenza vaccine nonresponders^34,35^. It is noteworthy that in NAFLD, low responders in addition to nonresponders show dysfunctional pTFHs. Studies have shown that prevaccination levels of CXCL11 and CXCL12 in healthy individuals and CXCL13 in HIV+ subjects may predict hepatitis B-vaccine response^34,36^. Thus, the results of our current study suggest that baseline CXCL13 could potentially be a biomarker for vaccine response in NAFLD, and an area for future investigation.

There is evidence regarding the role of human leukocyte antigen (HLA) alleles in response to HBV vaccination with HLA-DRB1 and HLA-DQB1 being associated with strong antibody responses^37^. We did not perform HLA typing in our study; however, we conducted parallel preclinical studies in a NAFLD mice model to account for vaccine responses within a model of identical genetic backgrounds. We noted blunted anti-HBs and T cell response in mice fed a HFD compared to normal weight mice on normal diet. In the first experiment, mice were fed HFD for 9 weeks and then immunized. This group recapitulates the cohort of NAFLD patients receiving the vaccine. In the second group, both animal groups were immunized at baseline and then fed either a normal vs. HFD, and hence potentially represents the human scenario of HBV vaccination in non-obese children who may subsequently develop adult onset obesity and NAFLD. In agreement with our results, Miyake et al.^33^ found impaired HBsAg-specific-humoral and T-cell responses in NAFLD mice who received a similar diet. It would be interesting to develop a mouse model that recapitulates nonalcoholic steatohepatitis (NASH) and study mechanism(s) of HBV vaccination response in NAFLD vs. NASH.

Our study did not include an age-matched healthy arm, however, a pooled analysis of previous studies in ~2000 healthy immunocompetent adults predicted >80% seroconversion rates in adults up to 60 years of age^28^. Additionally, we did not stratify patients with lean NAFLD (BMI < 25), low-risk (BMI: 25–29.9), medium-risk (BMI 30–34.9), and high-risk obesity cases (BMI > 35) or perform comparisons based on waist circumference. However, the current study of 68 adults is one of the largest reported to date regarding HBV vaccine responses in obese adult patients with confirmed diagnosis of NAFLD.

As per clinical guidelines and vaccine monographs, approaches for inducing vaccine response in non and low responders include repeating the vaccination series, a single booster dose or immunization with higher doses. The use of a two-dose novel vaccine, Heplisav-B^®^-induced high seroprotection rates in diabetics >70 years^38^. Although this vaccine is approved by the U.S. Food and Drug Administration (FDA), it is not yet approved by Health Canada. Whether NAFLD patients benefit from this vaccine is an interesting area of future investigation. It is known that 5–10% of healthy adults are nonresponders to the HBV vaccine, 15–50% of vaccine recipients are anti-HBs negative within 5–10 years after vaccination^39^. The results of the current study are remarkable in that 15% (10/68) of NAFLD patients were non-responders to the primary vaccination as early as 1-month post vaccination. Gara et al.^40^ determined that anti-HBs titers decline more slowly among at-risk adults compared to childhood vaccine recipients. Although a recent study showed vaccine-specific-T-cell responses lasting at ~20–30 years after adult immunization^41^, to date no studies have explored long-term humoral and adaptive immune response to the HBV vaccine in NAFLD adults. We plan to conduct a follow-up study to assess long-term responses (i.e., antibody waning, the persistence of T-cell immunity and anamnestic response) to the vaccine in individuals enrolled in this study.

In summary, this study showed significantly reduced HBV vaccine-specific-antibody and T cell responses in NAFLD patients with a BMI > 35 compared to low-risk obesity class NAFLD patients with BMI < 35. The hepatitis B vaccine is one of the most important human vaccines recommended worldwide. Obesity and NAFLD impairs normal immune functioning and may further perpetuate chronic disease development, and complications of the metabolic syndrome. This clinical study, together with complementary animal model data, can inform vaccine strategies, development of improved immunization protocols and highlight the impaired host immune response in high-risk obesity class individuals with NAFLD.

## Patients and methods

### Study participants and vaccines

Sixty-eight HBV naive NAFLD patients (18–60 years) in this study were recruited from specialized NAFLD clinics at five Canadian centers from August 2016–2019. Exclusion criteria included subjects <18 and >60 years of age, pregnancy, human immunodeficiency virus+, HCV+, decompensated cirrhosis, and those with serological evidence of HBV exposure or immunization (HBsAg, anti-HBs) and total HBV core antibody (anti-HBc). All patients provided signed informed consent under an approved ethics protocol (Conjoint Health Research Ethics Board REB16-0274) according to the principles of Good Clinical Practice and the Declaration of Helsinki. The study was registered on ClinicalTrials.gov (NCT02985450). The in-clinic review included identifying risk factors for NAFLD (obesity/hyperlipidemia/metabolic syndrome/type 2 diabetes) in the absence of significant alcohol consumption and/or an ultrasound showing confirmed steatosis. The diagnosis of NASH vs. NAFLD is based on histopathological criteria (i.e., steatosis, lobular inflammation, and hepatocellular ballooning)^8^ which requires a liver biopsy^42,43^. However, few study patients underwent liver biopsy due to invasive nature in the current study and was often not clinically indicated if patients had normal transient elastography. All adults received the standard three-dose regimen (0–6 months) of Engerix B^®^ (20 µg of HBsAg and 500 µg aluminum hydroxide, Glaxosmithkline, GSK) or a combined HBV/HAV vaccine (Twinrix^®^,GSK) as per Canadian immunization guidelines^44^. The vaccines were purchased by patients or health practitioners and administered as an intramuscular injection into the deltoid muscle of the nondominant arm by clinic nurse.

### Assessment of anti-HBs antibodies after vaccination

Anti-HBs titers were assessed in patients at 1–3 months after complete vaccination (i.e., three doses of Engerix B^®^ or Twinrix B^®^) by a chemiluminescent microparticle immunoassay (detection range, 0–1000 IU/L, Abbott Architect, Mississauga, ON, Canada). HBV seroprotection was defined as anti-HBs ≥ 10 IU/L after complete vaccination^45^.

### Isolation of peripheral blood mononuclear cells (PBMC)

PBMC were separated by density gradient centrifugation from ~20 mL of heparinized blood, and ~10^7^ cells/vial were cryopreserved. PBMC were only isolated at the coordinating center at baseline (i.e., before the vaccination) and post vaccination for assessment of cellular response.

### Assessment of circulating memory B and T cells

The proportion of memory B and T cells in PBMC at baseline and post vaccination was assessed using flow cytometry. Approximately, 5 × 10^5^ PBMC were stained for memory B-cell markers using anti-CD19, anti-CD45, and anti-CD27. Memory T cells were identified using anti-CD3, anti-CD4, anti-CD8, anti-CD56, anti-CD45RA, and anti-CCR7 using multi-parameter flow cytometry (BD FACS Canto II, Toronto, ON, Canada) Catalog and lot numbers of the antibodies used can be found in Supplementary Table 2. Prior to staining, cells were stained with fixable viability dye FVS 510. Antibodies were used at dilutions recommended by the vendor. Appropriate isotype and compensation controls were used and a minimum of 100,000 events was acquired. Data were analyzed using FACS DIVA and Flowjo v11 (Treestar Inc., San Carlos, CA).

### HBsAg-specific proliferation assays

Fresh (or cryopreserved in 4 patients) PBMC (~10^6^) were labeled with 1 µM carboxyfluorescein-diacetate-succinimidyl-ester (CFSE; BD Horizon, San Diego, CA) according to vendor’s protocol. Labeled PBMC were stimulated with 5 µg HBsAg (adw) (ARP, Waltham, MA) in RPMI 1640 with 10% FBS and 2 mmol/L glutamine. Anti-CD3 (1 µg/mL) and anti-CD28 (5 µg/mL) (BD Biosciences, San Jose, CA) stimulated cells served as a positive control. Unstimulated DMSO-treated cells were used as negative controls. Cells were cultured in triplicates and plates incubated at 37 °C with 5% CO_2_ for ~8 days. Cell proliferation was assessed on day 8. SI was calculated as % CFSE low cells in stimulated cells / % CFSE low cells in the unstimulated control^46^. SI > 3 was considered positive for HBsAg-specific proliferation. Cells were stained using the memory T-cell panel and analyzed by flow-cytometry.

### Peripheral follicular helper T cell responses (pTFH)

Baseline and post-vaccination PBMC were labeled with anti-CD4 and anti-CXCR5 antibodies. In addition, CD19+ and CD4+ CXCR5+ cells were sorted (>85% purity) using BD FACS ARIA II (Supplementary Fig. 4) and 30,000 cells were cocultured (1:1) for 5 days in the presence of 3.5 µg of HBsAg + 2 µg/mL anti-CD28. Staphylococcal enterotoxin B (Sigma, Oakville, ON, Canada) was used as a positive control. Cell culture supernatant and serum levels of CXCL13 and IL-21 were measured using enzyme-linked immunosorbent assay (ELISA) (R&D Systems Inc., Oakville, ON, Canada).

### HBV vaccination in a mice model of NAFLD

Mice were purchased from Jackson Inc. (Bar Harbour, ME) and housed at the University of Calgary in a pathogen-free facility. All experiments were approved by the University of Calgary animal care committee in accordance with the Canadian Council for Animal care (AC16-0040). C57BL/6 male mice (6–8 weeks old, *N* = 40;10/group), were fed either a normal chow vs. HFD (40% fat, 40% sucrose, Dyets Inc., Bethelem, PA)^47^ and administered 2 doses of Engerix-B^®^ (0.05 µg/g body weight) 2 weeks apart, intramuscularly, either after inducing NAFLD phenotype (N→V) or at baseline prior to HFD (V→N). The induction of NAFLD phenotype in mice was confirmed on liver histology. Briefly, liver lobes were harvested from NAFLD and control mice, fixed in formalin and embedded in paraffin. Sections were cut at 4 µm diameter and stained with hematoxylin and eosin. Slides were blinded and evaluated by a clinical pathologist to assess for liver steatosis, inflammation, and hepatocyte ballooning.

### Assessment of anti-HBs and T cell response in a mice model of NAFLD

Blood was collected before vaccination and every 2 weeks post booster via tail vein bleeding and by cardiac puncture at the final time point. Whole blood was collected in heparinized tubes and spun at 1000*g* for 10 min to obtain plasma. Anti-HBs levels in control and HFD mice were determined using a qualitative ELISA (Aviva Biosystems Biology, San Diego, CA). Spleens were harvested at the final time point for lymphocyte proliferation assessment. A single-cell suspension of splenocytes was prepared and CFSE labeled as described above and ~10^6^ cells were stimulated with 5 µg of HBsAg. Concanavalin A (Con-A) (4 µg/mL) stimulated and DMSO treated cells served as positive and negative controls, respectively. On day 4–5, splenocytes were assessed for the proliferation of CD3+ CD4+ T cells using flow-cytometry (Supplementary Table 2).

### Statistical analysis

Data were analyzed using Graphpad Prism 7 (La Jolla, CA). Demographic, clinical, and laboratory parameters were compared using measures of central tendency; Mann–Whitney or ANOVA were used to compare anti-HBs titers; Mann–Whitney *U* test and Kruskal–Wallis test with post hoc Dunn’s test for multiple comparisons was used to compare proliferation and immunophenotyping data. Nonparametric Spearman’s rank correlation test was used for correlation analysis; *p* values < 0.05 were considered

### Reporting summary

Further information on research design is available in the Nature Research Reporting Summary linked to this article.

## Supplementary information

Supplementary Information

Reporting Summary

## Data Availability

All the relevant information is in the paper in the form of tables, figures, and supplementary material.
