# Supplementary material for: Reduced immune responses to hepatitis B primary vaccination in obese individuals with nonalcoholic fatty liver disease (NAFLD)
Source: NPJ Vaccines. 2021 Jan 11;6:9. doi: 10.1038/s41541-020-00266-4 (PMC7801497; doi:10.1038/s41541-020-00266-4)
Supplement: Supplementary file 1 — Supplementary Information [file 41541_2020_266_MOESM1_ESM.pdf]

Supplementary Table 1: Clinical Data of Patients Who Underwent Liver Biopsy

ID/Age/Sex	Fibroscan (Transient elastography), kPa	Fibrosis score	Controlled Attenuation Parameter (CAP) score, dB/m	NAFLD Activity Score (NAS) (Steatosis + Inflammation +Ballooning)	Anti-HBs (IU/L)
4139-0001/47/F	27.4	4	359	4#	1
0002-2081/53/F	17.5	2	NA	3 (2+1+0)	1.6
0021-2898/52/F	12	2	NA	6 (3+2+1)	4.7
0028-8096/56/F	50.5	4	400	6 (3+1+2)	158.6
0011-9100/43/M	16.9	3	183	3#	7
0032-5927/50/F	21.3	3	326	5 (2+2+1)	507.4
0033-3513/24/M	8.8	2	400	5#	1000
8931-0005/59/M	22.3	3-4	400	NA	36

NA, not available, # breakdown of the NAS score not available

Supplementary Table 2: List of antibodies used for flow cytometry in the study

Antibody used	Fluorochrome	Clone	Catalog #	Lot#
Mouse anti-human CD19	Brilliant Blue 515	HIB 19	564456	6014888
Mouse anti-human CD45	PE-Cy7	H130	557748	6091811
Mouse anti- human CD27	Alexa Fluor 647	M-T271	565116	9106651
Mouse anti- human CD3	Brilliant Violet 605	SK7	563219	5083720
Mouse anti-human CD4	Alexa Fluor 700	SK3	566321	8338738
Mouse anti-human CD8	APC-H7	SK1	560179	6140812
Mouse anti- human CD56	Brilliant Violet 421	NCAM 16.2	562751	6007717
Mouse anti- human CD45RA	PE-Cy7	HI 100	562885	6231513
Hamster anti-human CCR7	Alexa Fluor 647	3D12	565867	6070895
Rat anti-human CXCR5	Alexa-Fluor 647	RF8B2	558113	7306668
Mouse anti-human CD4	PerCP	SK3	566321	8338738
Rat anti-mouse CD3	Brilliant Violet 605	17A2	564009	8059759
Rat anti-mouse CD4	APC-H7	GK1.5	560181	8353964

Supplementary Figure 1

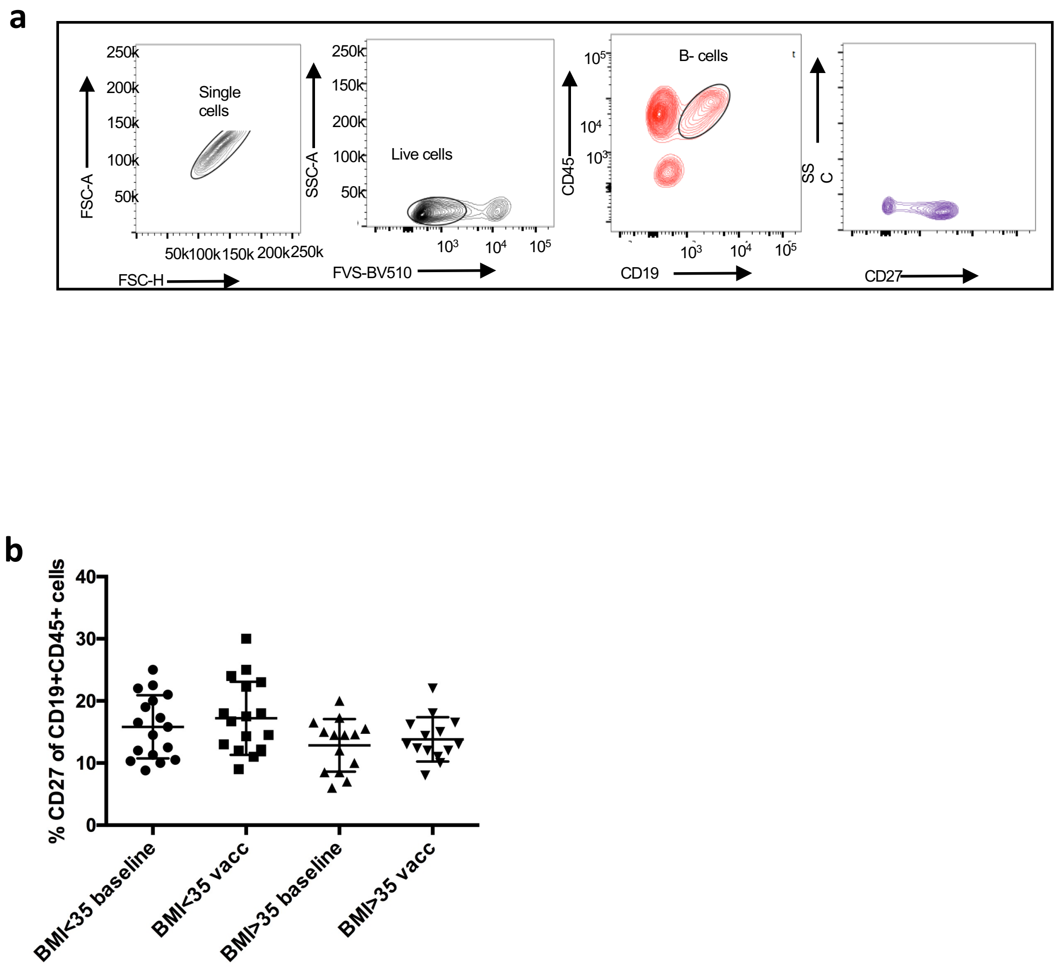

Analysis of memory B cells in NAFLD patients at baseline and after vaccination. (a) Gating strategy for memory B cells defined as CD45+CD19+CD27+ cells. (b) Frequency of circulating memory B-cells at baseline and after vaccination was comparable (Krushkal-Wallis test, $p>0.05$); vacc, vaccination

Supplementary Figure 2

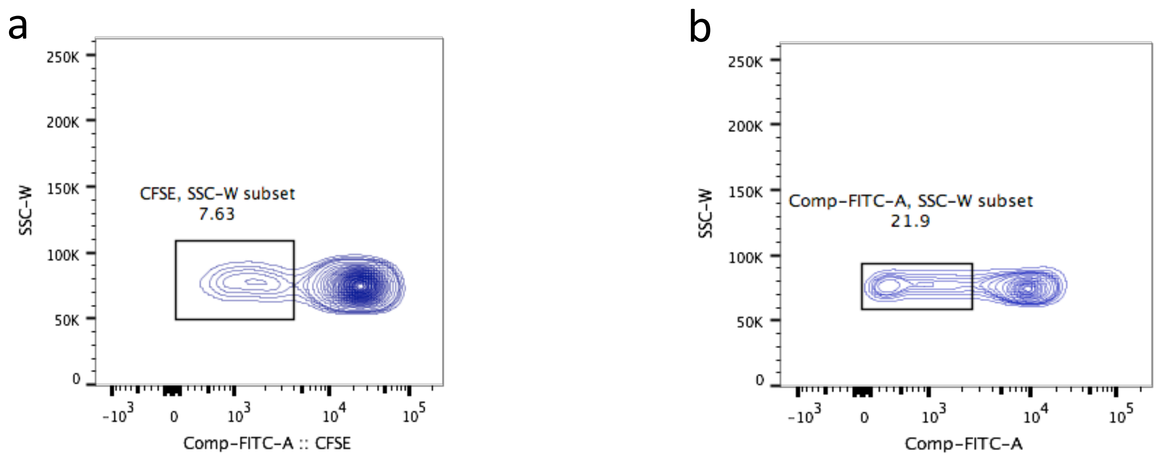

Representative figures showing proliferation of CD8+ T cells in a low risk (a) and high risk obesity class case (b)

Supplementary Figure 3

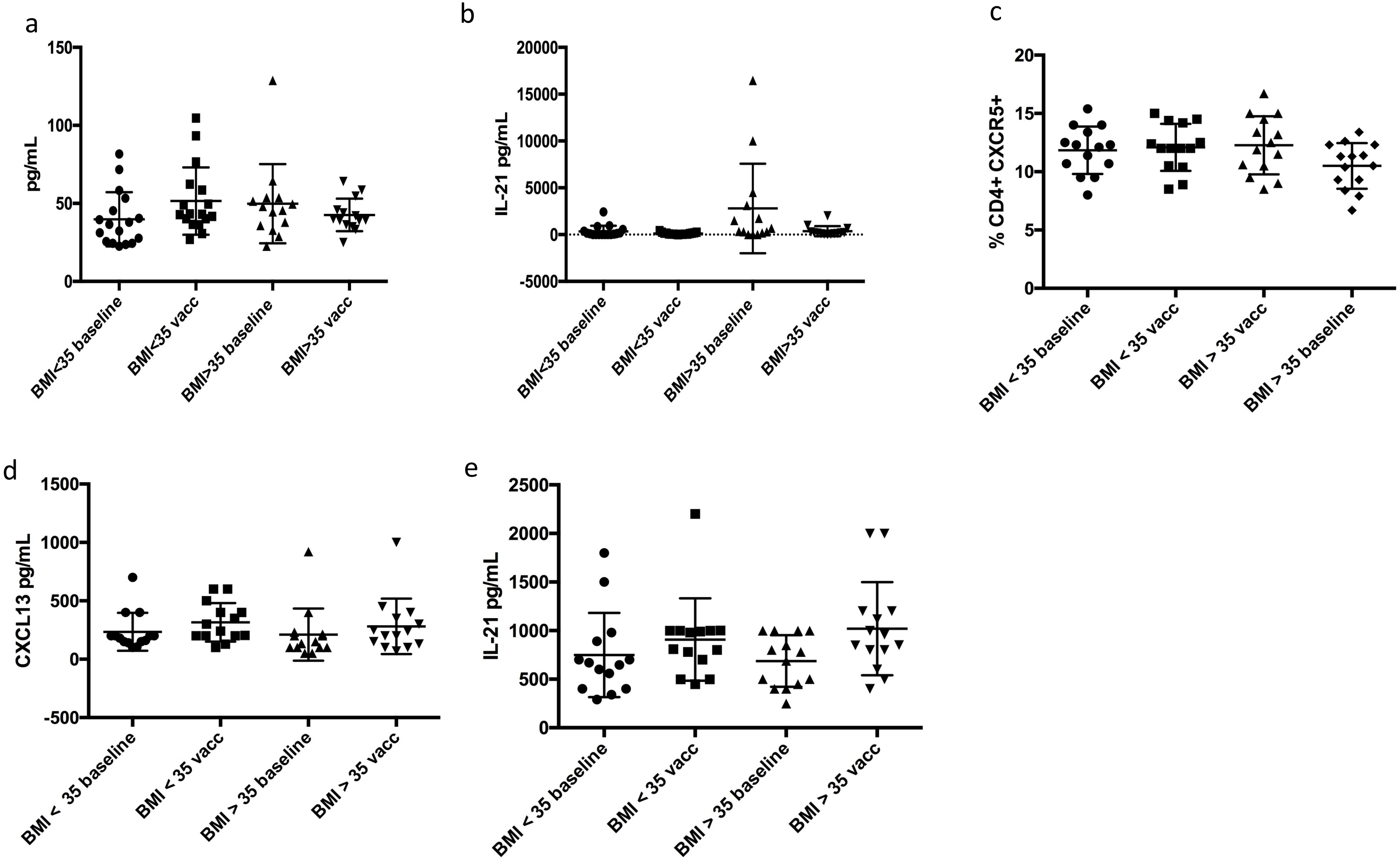

a. Serum CXCL13 b. IL-21 c. % pTFH cells in NAFLD patients with BMI<35 and BMI>35 at baseline vs. vaccination d. HBsAg specific CXCL13 levels e. IL-21 levels in co-cultured cell supernatants in NAFLD patients with BMI< 35 and BMI>35

Supplementary Figure 4

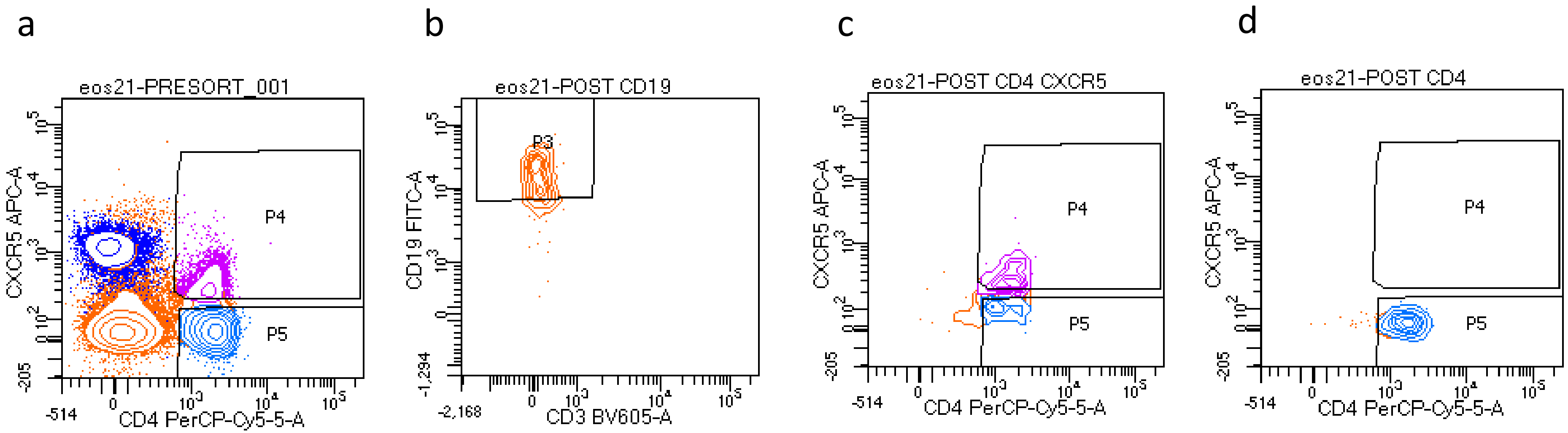

Representative plots of CD4+CXCR5+ cells, (a) pre and (b,c,d) post sort
